# Enhanced Mechanical and Hydrophobic Antireflective Nanocoatings Fabricated on Polycarbonate Substrates by Combined Treatment of Water and HMDS Vapor

**DOI:** 10.3390/ma16103850

**Published:** 2023-05-19

**Authors:** Yao Yan, Jia Liu, Bing Zhang, Ruohan Xia, Yuqi Zhang, Zisheng Guan

**Affiliations:** College of Materials Science and Engineering, Nanjing Tech University, 30 South PuZhu Road, Nanjing 211816, China; 202061203187@njtech.edu.cn (Y.Y.); 202061203272@njtech.edu.cn (J.L.); 202061203152@njtech.edu.cn (B.Z.); 202061203203@njtech.edu.cn (R.X.); 202161203205@njtech.edu.cn (Y.Z.)

**Keywords:** antireflective nanocoatings, silica, mechanical stability, hydrophobicity, polycarbonate

## Abstract

Polycarbonate (PC) with high transmittance, stable mechanical performance and environmental resistance is crucial for practical applications. In this work, we report a method for the preparation of a robust antireflective (AR) coating by a simple dip-coating process of a mixed ethanol suspension consisting of tetraethoxysilane (TEOS) base-catalyzed silica nanoparticles (SNs) and acid-catalyzed silica sol (ACSS). ACSS greatly improved the adhesion and durability of the coating, and the AR coating exhibited high transmittance and mechanical stability. Water and hexamethyldisilazane (HMDS) vapor treatment were further employed to improve the hydrophobicity of the AR coating. The as-prepared coating exhibited excellent antireflective properties, with an average transmittance of 96.06% in the wavelength range of 400 to 1000 nm, which is 7.55% higher than the bare PC substrate. After sand and water droplet impact tests, the AR coating still maintained enhanced transmittance and hydrophobicity. Our method shows a potential application for the preparation of hydrophobic AR coatings on a PC substrate.

## 1. Introduction

Polycarbonate (PC) has become one of the most widely used optical plastic materials because of its high transmittance, outstanding impact resistance, good temperature resistance and excellent toughness [1,2,3,4]. For shatterproof glass, eyeglasses, optical lenses and other optics that need to improve sunlight transmittance while reducing sunlight reflection, one of the best methods to increase the effectiveness of light usage is to apply antireflective (AR) coatings on the surface of optical components [5,6,7]. Different physical and chemical techniques have been employed to prepare antireflective coatings, including chemical vapor deposition [8], vacuum vapor deposition [9], nanoscale etching [10], layer-by-layer assembly [11] and sputtering [12]. However, the application of AR coatings is limited by the particular equipment, environmental requirements and intricate operating procedures required for these methods. In contrast, dip-coating is a simple and low-cost method for the preparation of AR coatings [13,14]. In addition, AR coatings are difficult to apply in daily life due to suboptimal mechanical stability (the coating cracking or peeling from the substrate surface under external forces) and environmental resistance (dust, humidity and brine corrosion, among others) [15]. To adapt to different application scenarios, many durable AR coatings have been produced through surface modification and structural optimization. Zhang et al. partially embedded the silica coating into the PC substrate via chloroform vapor treatment to enhance the adhesion [16]. The chloroform divided the silica coating into a base layer and an intermediate layer with different refractive indices to achieve antireflection. Adachi et al. prepared a photocatalytic TiO_2_/SiO_2_ coating with excellent superhydrophilic wettability on a lightweight polycarbonate substrate [17]. Ai et al. prepared mesoporous silica coatings (MSCs) using UV-O_3_ exposure and ammonia vapor treatment of dried poly (ethylene glycol)-block-poly (propylene glycol)-block-poly (ethylene glycol) (F127)-containing acidic silica gel [18]. The prepared MSCs displayed scrub resistance (400 scrubbing cycles with a brush under a load of 4.5 N) comparable to that of coatings that had been heated to 250 °C. However, these operating procedures, involving special equipment and complex conditions, limit the application of AR coatings. Among various coating preparation processes, the sol-gel method shows some great advantages, such as a low-temperature preparation process, excellent coating uniformity, a simple process and an adjustable refractive index [19,20,21,22]. Fan et al. prepared a hydrophobic double-layer tri-wavelength broadband AR coating by modifying the base-catalyzed SiO_2_ film with ammonia and methyltriethoxysilane (MTES) vapor with high transmittance values of 99.60%, 98.85% and 99.16% at 355 nm, 532 nm and 1064 nm, respectively. The modified coating exhibits good optical stability in humid environments [23]. Guo et al. prepared SiO_2_-TiO_2_ closed-surface antireflective coatings by adding TiO_2_ nanoparticles to a nanocomposited silica sol consisting of acid-catalyzed nanosilica networks and silica hollow nanospheres (HNs). The SiO_2_-TiO_2_ closed-surface ARC shows high transmittance and excellent reliability in high-moisture conditions [24]. Esfahani et al. prepared silica thin films with antireflective, hydrophobic and anti-icing properties on glass substrates by adding trichloromethyl silane (TCMS) to silica sols based on TEOS (tetraethoxysilane) and TMMS (trimethoxymethylsilane) precursors. The silica films exhibited high transparency (94.59%) and hydrophobicity (110 ± 1°) [25]. The Stöber method is commonly employed for the fabrication of silica nanocoatings. The coatings prepared by the Stöber base-catalyzed method are porous and exhibit great transmittance. However, the silica nanoparticles (SNs) in the coatings are only connected by point-contact forces. Therefore, the bonding force of the coating is very weak. In contrast, the growth process of ACSS produces a structure of linear chains, which results in a strong adhesion force, and, therefore, the coatings prepared by ACSS show enhanced mechanical stability [26].

Aside from mechanical performance, environmental resistance is the key to the practical application of silica AR coatings. Due to the nanopore structure of silica nanocoating and the abundance of Si-OH groups on the surface, ambient moisture and organic pollutants can easily adsorb on the silica coating, leading to the degradation of its optical properties [27,28]. The environmental resistance of AR coatings can be greatly enhanced by introducing hydrophobic groups [29].

In this paper, we report a simple and low-cost method to prepare antireflective coatings with high transmittance and enhanced mechanical robustness using tetraethoxysilane (TEOS) base-catalyzed silica nanoparticles (SNs) mixed with acid-catalyzed silica sol (ACSS). Furthermore, in order to improve the hydrophobicity and environmental resistance of the silica nanocoatings, AR coatings on PC substrates were modified sequentially with water and hexamethyldisilazane (HMDS) vapor to cover the polar hydroxyl groups on the surface of the coating with non-polar methyl groups. To evaluate the stability of the coatings, a sand impact test [30], water droplet impact test [31], easy-clean test [32] and environmental resistance (humid environment and brine corrosion) test [29,33] were performed. The results indicated that the as-prepared AR coatings possessed stable mechanical performance and environmental resistance.

## 2. Experimental Procedure

### 2.1. Materials

PC (60 mm × 25 mm × 2 mm) was used as the substrate and was obtained at the local market. Tetraethoxysilane (TEOS, ≥99%), hexamethyldisilazane (HMDS, 98%) and Sodium chloride (NaCl, 99.5%, AR) were purchased from Sinopharm Chemical Reagent Co. (Shanghai, China). Ammonia (NH_3_·H_2_O, 25%, AR), hydrogen peroxide (H_2_O_2_, 30%, GR), anhydrous ethanol (C_2_H_5_OH, EtOH, 99.5%, AR) and concentrated hydrochloric acid (HCl, 37%) were purchased from Shanghai Lingfeng Chemical Reagent Co. (Shanghai, China). The soil powder was homemade in the laboratory. The water was extremely pure deionized water with a resistivity of 18.25 MΩ·cm. All the chemical reagents were employed directly without additional purification.

### 2.2. Preparation of SNs

Silica nanoparticles (SNs) were synthesized by the base-catalyzed Stöber method. A total of 50 mL of absolute ethanol and 2.5 mL of aqueous ammonia were mixed together by stirring in a beaker. The beaker was then placed in a 60 °C water bath, and 3 mL of TEOS was added after the temperature stabilized. After stirring for 10 h, the ethanolic suspension of SNs was prepared. The resultant sol was stirred in a fume hood to remove ammonia in the suspension and form a stable sol. Finally, the silica sol was aged for 5 days in a sealed glass container at 25 °C. The average particle diameter of silicon nanoparticles is approximately 30 nm. Assuming that 1 mole of TEOS completely reacts to produce 1 mole of silica, the theoretical concentration of silica in this sol was calculated as 50.65 mg/mL.

### 2.3. Synthesis of ACSS

Acid-catalyzed silica sol (ACSS) was prepared in accordance with a reference with slight modifications [34]. TEOS (5 mL) was mixed with ethanol (50 mL), concentrated HCl (0.02 g) and water (1.67 mL). The solution was stirred at 30 °C for 4 h and then aged at room temperature for 3 days to allow the complete reaction of TEOS. The theoretical concentration of silica in this sol was calculated as 82.67 mg/mL.

### 2.4. Preparation of Mixed Suspensions

ACSS was injected into the SNs while being stirred magnetically at room temperature to create a succession of SNs@ACSS suspensions when the silica sols were properly aged. The volume ratios of SNs/ACSS were 1.0, 2.0, 3.0, 4.0 and 5.0, respectively. All the suspensions were stirred for 30 min at room temperature.

### 2.5. Preparation of Antireflective and Hydrophobic Coatings

**Figure 1:**
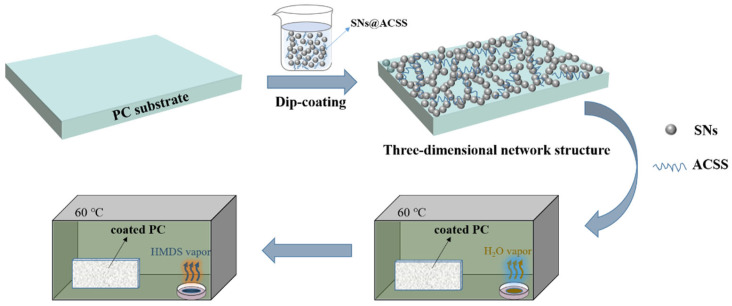
Schematic diagram of the preparation of robust AR coating from SNs@ACSS mixed suspension and the subsequent water and HMDS vapor treatment processes.

PC substrates were pretreated with hydrogen peroxide before dip-coating. The cleaned PC was treated in a hydrothermal kettle containing H_2_O_2_ at 50 °C for 0.5 h to increase the adhesion between the coating and the substrate. All the AR coatings were prepared by the dip-coating method on both sides of the PC substrate. Figure 1 shows the fabrication process of the SNs@ACSS coatings. The PC substrates were dipped vertically into a mixture of base-catalyzed silica sol and ACSS with a lifting rate of 100 mm/min and an immersion time of 120 s and then dried at room temperature.

The HMDS modification was applied to the AR coating to increase the hydrophobicity of the silica nanocoatings. The effects of water and HMDS vapor treatment and HMDS soaking treatment on the transmittance and hydrophobicity of the SNs@ACSS coatings were compared. The coated PC substrates were treated with water vapor for 30 min at 60 °C in a 1000 mL thermostat containing 10 mL of deionized water in the water and HMDS vapor treatment processes (Figure 1). Then, the samples were further treated with HMDS vapor for 60 min at 60 °C in a 1000 mL thermostat containing 10 mL of HMDS. The HMDS soaking treatment involved soaking the coated PC in a 15 wt.% solution of HMDS hexane for 15 min. After the water and HMDS vapor treatment and HMDS soaking treatment, the SNs@ACSS coatings were named SNs@ACSS-HD and SNs@ACSS-HS, respectively.

### 2.6. Characterization

The microstructure morphology of the coatings was observed by scanning electron microscopy (SEM, JEOL, JSM-IT200) and field emission scanning electron microscopy (FESEM, Hitachi (Tokyo, Japan), 4800) at an accelerating voltage of 5 kV. The Fourier transform infrared (FTIR) spectra of the pure silica and HMDS-modified silica were measured on a Thermo Scientific Nicolet iS20 spectrometer. The transmission spectra in the wavelength range of 300 to 1100 nm were recorded using a UV-Vis spectrophotometer (UV-vis, UV-2600, Shimadzu (Tokyo, Japan)). Atomic force microscopy (AFM, Bruker (Billerica, MA, USA)) was used to characterize the surface roughness and obtain three-dimensional surface images of the coating. The scanning area was 10 μm × 10 μm. The water contact angles (WCAs) on the coated PC were measured at ambient temperature with a contact angle goniometer (JC2000CS). The water contact angles were measured at five different areas of each sample surface for error estimation.

## 3. Results and Discussion

### 3.1. Effect of SNs/ACSS Volume Ratio on Transmittance

**Figure 2:**
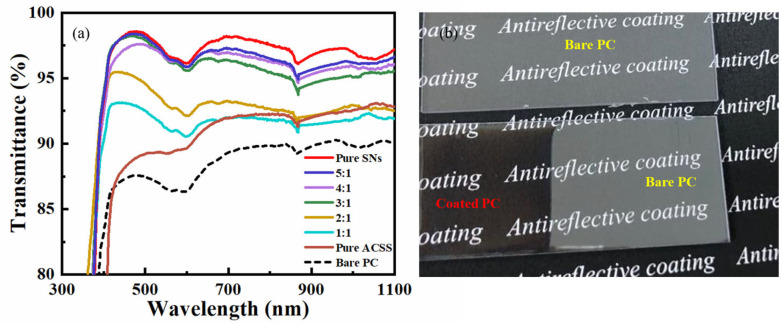
(**a**) Transmission spectra of the various coatings fabricated by different volume ratios of SNs and ACSS; (**b**) Digital images of the bare PC and SNs@ACSS coated PC.

By adjusting the volume ratio of the SNs and ACSS, a number of mixed suspensions were produced. Figure 2a shows the SNs/ACSS (volume ratio) effect on the transmittance of the silica nanocoatings. As the SNs/ACSS increased from 1:1 to 5:1, the transmission spectra demonstrated that the transmittance of the coated PC gradually increased. When the SNs/ACSS was 1:1, the coated PC transmittance was only slightly increased compared to the bare PC, with an average transmittance of 91.80%. As the SNs/ACSS increased from 2:1 to 3:1, the transmittance of the coated PC increased significantly, with an average transmittance of 96.03% and a maximum transmittance of 98.24%. By further increasing the proportion of SNs, the transmittance of the coated PC was not significantly increased. The results showed that the proper introduction of ACSS has little effect on the transmittance of silica nanocoatings. The introduction of excessive ACSS led to the aggregation of silica nanocoatings and the formation of inhomogeneous nanocoatings, which resulted in only a small increase in the transmittance.

The stability of nanocoatings is crucial for their practical application [30,35]. The growth pattern of silica sol is skewed toward spherical swelling particles in the presence of an alkaline catalyst. Base-catalyzed silica particles are porous and spherical, resulting in the formation of films with very low refractive indices, while acid-catalyzed silica is in the form of chains. The ACSS can help create a strongly cross-linked silica network by linking together silica nanocoating as the acid-base mixed sol-gel coating, thereby significantly improving the robustness of the coating. Therefore, considering the transmittance and stability of the coating, an SNs/ACSS volume ratio of 3:1 was the optimal ratio for the next test. The AR coating was named SNs@ACSS. The maximum transmittance of the SNs@ACSS coated PC was about 98.24% at 470 nm, while the maximum transmittance of the bare PC was only 90.28%. A digital image taken under room sunlight made it easy to observe the related antireflective characteristic of the coated PC with increased transmittance. The SNs@ACSS coated PC significantly reduced light reflection compared to the bare PC, as shown in Figure 2b.

### 3.2. Surface Morphology, Transmittance and Wettability of As-Prepared Coatings

**Figure 3:**
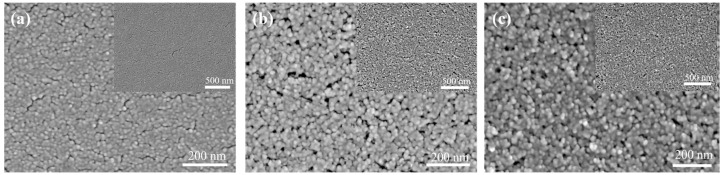
SEM micrographs of surface. (**a**) Pure SNs nanocoating; (**b**) SNs@ACSS coating; (**c**) SNs@ACSS-HD coating.

Figure 3 shows the surface morphology of the coatings characterized by FESEM. For the nanocoating prepared with pure SNs (Figure 3a), the surface of the silicon nanocoating was relatively flat. Due to the fact that the silica nanoparticles (SNs) in the coating were only connected by point-contact forces [26], the surface of the nanocoating had many cracks. After the introduction of ACSS (Figure 3b), the silica nanocoatings were interconnected compared to the coatings prepared with pure SNs. The surface of the coating had become less flat, which may have led to a slight decrease in the transmittance. The addition of an appropriate amount of linear ACSS can make the SNs highly cross-linked and form a three-dimensional network structure. This can greatly strengthen the adhesion of the SNs to one another and the substrate, thereby improving the mechanical robustness of the SNs@ACSS coatings. Figure 3c shows the surface morphology of the coating after modification with water and HMDS vapor. The surface structure of the coating was not significantly altered. This explained the almost constant transmittance of the coating after the modification.

**Figure 4:**
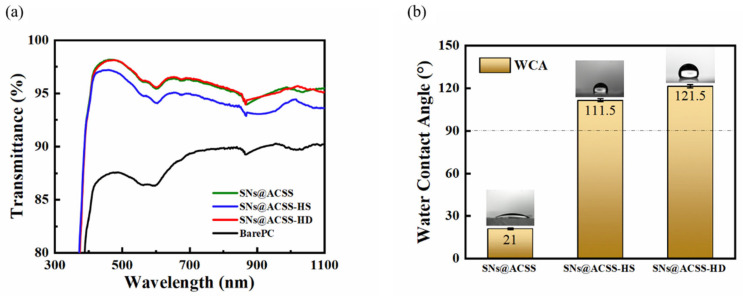
(**a**) Transmission spectra and (**b**) Water contact angles of the SNs@ACSS-coated PC (unmodified), SNs@ACSS-HS-coated PC (HMDS soaking treatment) and SNs@ACSS-HD-coated PC (water and HMDS vapor treatment).

Due to the hydroxyl and porous structure of the silica coating surface, water and contaminants in the environment can easily penetrate into the nanopores of the coating, resulting in a higher refractive index of the SNs@ACSS coating. Researchers introduced hydrophobic groups to promote stable applications of AR coatings in optics [36]. In this paper, water and HMDS vapor treatment were used to modify the AR coatings in order to maintain the high transmittance of the coatings while improving their environmental resistance. Figure 4 shows the effect of HMDS vapor treatment and HMDS soaking treatment on the transmittance and water contact angle of the SNs@ACSS coating. Figure 4a shows the transmittance of the SNs@ACSS-HD-coated PC and SNs@ACSS-HS-coated PC. The average transmittance of the SNs@ACSS coating soaked in the HMDS hexane solution decreased significantly from the unmodified state to the modified state, falling from 96.01% to 94.72% in the wavelength range of 400 to 1000 nm. The transmittance of the SNs@ACSS-HD coating, in comparison, remained constant and even slightly increased after being treated with water and HMDS vapor. The ethoxy groups in the silica coating that have not yet been fully hydroxylated can be further hydrolyzed by water vapor treatment, leading to the hydroxylation of the coating surface. These hydroxyl groups served as reaction poles in the subsequent HMDS vapor treatment and were swapped out for hydrophobic methyl groups. This significantly reduced the surface energy of the AR coating and improved the hydrophobicity of the silica nanocoating. Figure 4b shows the water contact angles of the coatings. The water contact angle increased from 21° to 121.5° after water and HMDS vapor treatment, while the contact angle of the AR coating directly soaked in a 10% HMDS hexane solution was only 111.5°. These findings suggested that water and HMDS vapor treatment can improve the hydrophobic properties of the AR coatings without decreasing the transmittance of the coatings.

**Figure 5:**
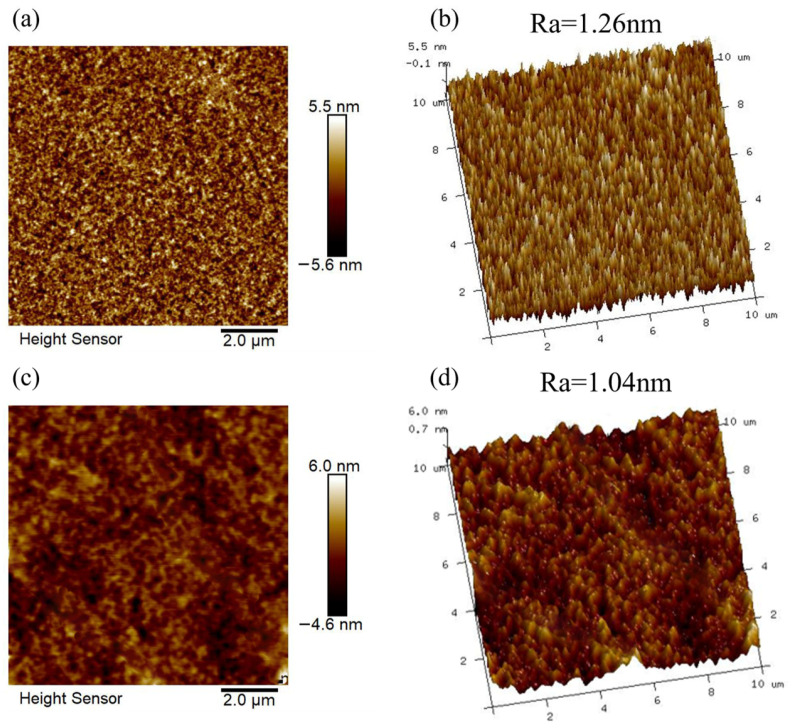
AFM surface topography and 3D images of the SNs@ACSS coating (**a**,**b**) and SNs@ACSS-HD coating (**c**,**d**).

Tadanaga [37], Li [38] and Sun [39] et al. demonstrated that if only the roughness is altered, at least 50 nm of roughness is necessary to prepare hydrophobic coatings. However, high surface roughness can lead to a high light absorption coefficient and reduce the antireflective properties of the coating. Figure 5 shows the AFM images of the SNs@ACSS coating before and after water and HMDS vapor treatment. The silica coating was loose and porous before the modification, and there was significant surface roughness, as shown in Figure 5a,b. The surface of the modified coating became flat, which reduced the diffuse reflection of the coating surface (Figure 5c,d). The surface roughness (Ra) of the AR coatings before and after HMDS modification were 1.26 and 1.04, respectively. This illustrated why the SNs@ACSS-HD-coated PC has a higher average transmittance than the unmodified coated PC.

### 3.3. Composition of Nanocoatings

**Figure 6:**
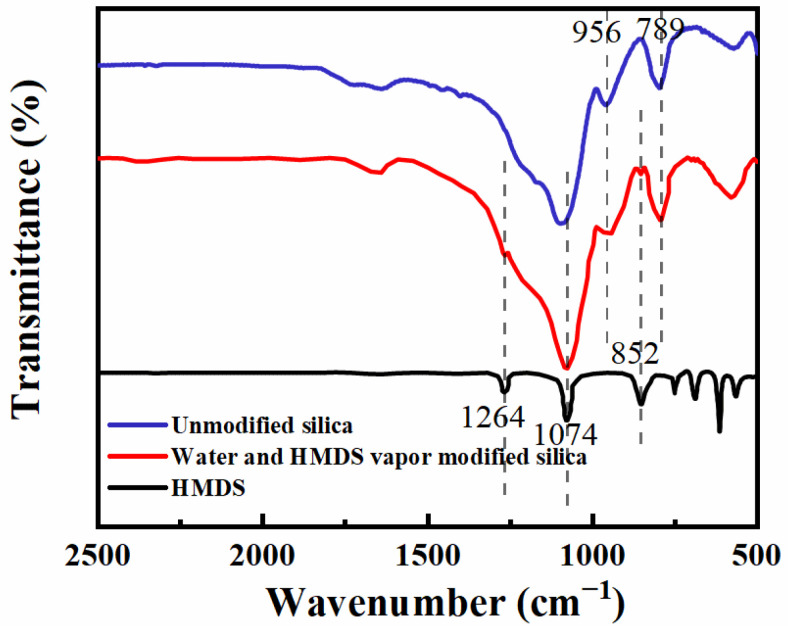
FTIR spectra of HMDS, unmodified silica and water and HMDS vapor modified silica.

The structure and chemical composition of the coatings have a significant impact on their performance. The hydrophobicity of the AR coating that was treated with water and HMDS vapor increased significantly, which was attributed to the substitution reaction of non-polar hydrophobic methyl groups to replace the polar hydroxyl groups on the coating, resulting in a significant reduction in the surface energy of the SNs@ACSS coating. FTIR spectroscopy revealed several characteristic absorption bands between pure silica as well as water and HMDS vapor-modified silica, as shown in Figure 6. These bands demonstrate that the methyl groups were successfully grafted onto the SiO_2_ surface. It can be observed that there is a strong absorption peak at 1074 cm^−1^ that corresponds to the stretching mode of Si-O-Si bonds [29]. The absorption band at 956 cm^−1^ corresponds to the hydroxyl group of Si-OH, and the intensity of this band was significantly weakened by HMDS vapor modification [40]. New absorption bands at 1264 and 852 cm^−1^ were discovered by contrasting pure silica with water and HMDS vapor-modified silica coatings. These bands were attributed to Si-C stretching vibration and Si-CH_3_ symmetric deformation, indicating the presence of -CH_3_ groups in the modified coatings [26]. These findings demonstrate that the polar -OH groups on the surface of the SNs@ACSS coating were successfully replaced by the hydrophobic -CH_3_ groups in HMDS.

### 3.4. Easy-Clean Property

**Figure 7:**
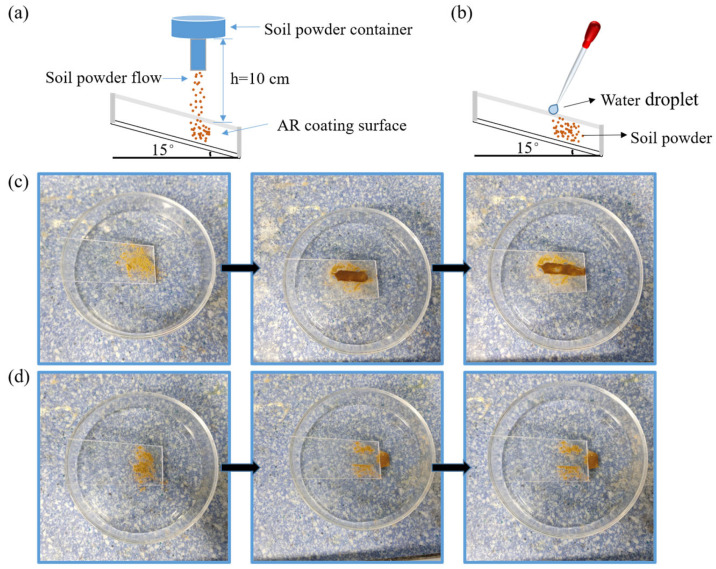
Easy-clean test of the AR coating. (**a**) Schematic diagram of soil powder spillage; (**b**) Schematic diagram of easy-clean test; (**c**) Bare PC surface; (**d**) SNs@ACSS-HD-coated PC surface.

The surface of transparent substrates is susceptible to contamination, resulting in a significant loss of transmittance [32]. Being able to be easily cleaned, often referred to as the ‘easy-clean’ property for coatings, is equally important for AR coatings. Hydrophobicity usually gives the coating easy-clean properties. In this work, the hydrophobic coating was tested for ease of cleaning using soil powder as a contaminant. As shown in Figure 7a, approximately 100 mg of soil powder was evenly sprinkled on the surface of the coated PC from a height of 10 cm in 10 s. The sample was inclined at an angle of 15°, and the concentration area of the soil powder was approximately 2 cm × 2 cm. As shown in Figure 7b, the water droplets (0.2 mL water) produced by the dropper slowly impinged onto the contaminated surface. Figure 7c,d shows the movement of the water droplets on the bare PC and the SNs@ACSS-HD-coated PC surface, respectively. As shown in Figure 7c, the water droplet impacted the bare PC surface and adhered to the PC surface along with the soil powder. Conversely, as shown in Figure 7d, the water droplets carried away contaminants from the SNs@ACSS-HD-coated PC surface due to the hydrophobic properties of the coating. This result shows that the coating modified by HMDS has certain easy-clean properties.

### 3.5. Mechanical Robustness of Nanocoatings

The stability of the nanocoatings in practical applications is determined by the mechanical robustness of the AR coatings. Due to the insufficient adherence between nanocoatings and substrates, AR coatings tend to lose their antireflective and hydrophobic properties when exposed to invasive contact. It has been demonstrated that linear silicate-like polymers made from acid-catalyzed silica sols serve as an inorganic binder [41,42]. In this work, the addition of ACSS contributed to the creation of a highly cross-linked silica network, and the SNs were connected to each other by strong Si-O-Si bonds, which greatly enhanced the mechanical stability of the coatings.

**Figure 8:**
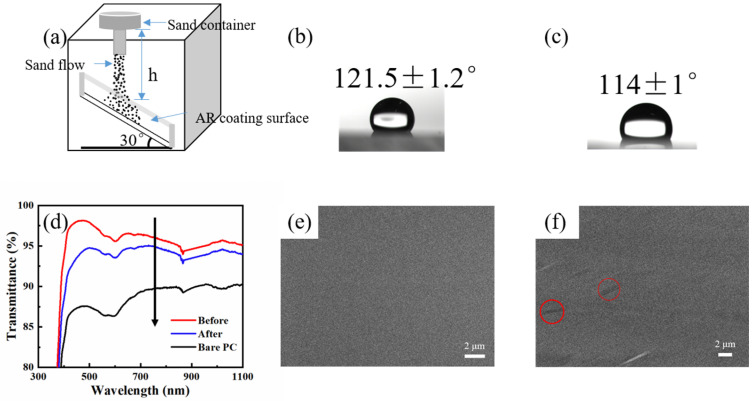
(**a**) Schematic diagram of the sand impact test; (**b**) WCA before the test; (**c**) WCA after the test; (**d**) Transmission spectra before and after the sand impact test; SEM images of the coating before (**e**) and after (**f**) the sand impact test.

In this study, the mechanical stability of the as-prepared hydrophobic AR coating was evaluated by the sand abrasion test as well as the water droplet impact test. Figure 8 shows the wettability, transmission spectra and surface morphology of the SNs@ACSS-HD-coated PC before and after the sand impact test. The test conditions were more demanding than in previous work, with higher initial heights and heavier sand masses [43]. A total of 50 g of sand was impacted on both sides of the PC from a height of 100 cm in 30 s, and the particle size of the sand was 100–300 μm. The average transmittance in the wavelength range of 400 to 1000 nm decreased from 96.06 to 94.04% with a reduction of 2.02%, as shown in Figure 8d, and the water contact angle decreased from 121.5 to 114°. Obviously, the as-prepared coating still maintained its excellent antireflective and hydrophobic properties. Figure 8e,f shows the SEM images of the coating before and after the sand impact test. The silica nanocoatings were slightly scratched off after the test, and only a small portion of the nanostructure was destroyed. The antireflective property and hydrophobicity of the coating were slightly decreased. However, the main coating on the PC substrate still provided the coating with hydrophobicity and antireflective property. 

**Figure 9:**
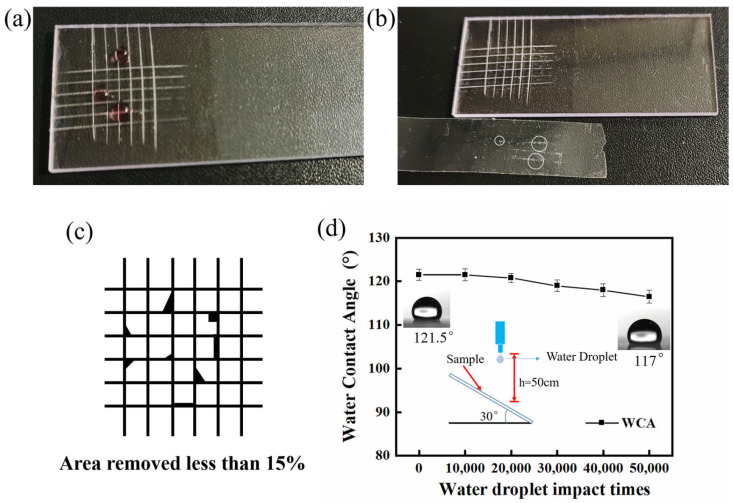
(**a**) Digital image and wettability of SNs@ACSS-HD AR coating after scratch cutting; (**b**) Digital image and the test result of antireflective coating after tape adhesion test; (**c**) Schematic diagram of the adhesion test result; (**d**) Water contact angles (WCAs) of the coating before and after 50,000 times water droplet impact test.

The tape adhesion test was used to assess the adhesion of the antireflective hydrophobic coating [44]. Figure 9a shows the digital image of the coating surface after scratch cutting and the wettability of the coating after cutting. As can be seen from the figure, the coating is still hydrophobic. Figure 9b shows the digital image of the coating after 3M tape adhesion and the tape adhesion test result. Only the coating on the edge of the scratch has partially peeled off. Figure 9c shows the results of the adhesion test. Less than 15% of the nanocoating was removed after the 3M tape adhesion test. The adhesion level of the coating was classified as class 3B according to the ASTM D3359 tape adhesion test. In addition, the water droplet impact test was also conducted to assess the surface durability of the coating [31]. In this test, approximately 50,000 water droplets fell freely from a height of 50 cm above the coated PC substrate, and the droplets collided with the substrate at 60°. The water droplet diameter was approx. 5 mm. Figure 9d shows the change in the contact angle of the coating surface before and after the water droplet impact test. The WCAs of the coating gradually decreased during the impact of the water droplets. After 50,000 water droplets impacted, the WCA decreased from 121.5° to 117°, and the nanocoating remained hydrophobic. These characteristics are extremely critical for the practical application of AR coatings. Inorganic silica nanocoatings are bound together by ACSS to create a strongly cross-linked silica network. This increases the mechanical resistance of the coatings while enabling the nanocoatings to withstand a high impact force.

### 3.6. Environmental Resistance

**Figure 10:**
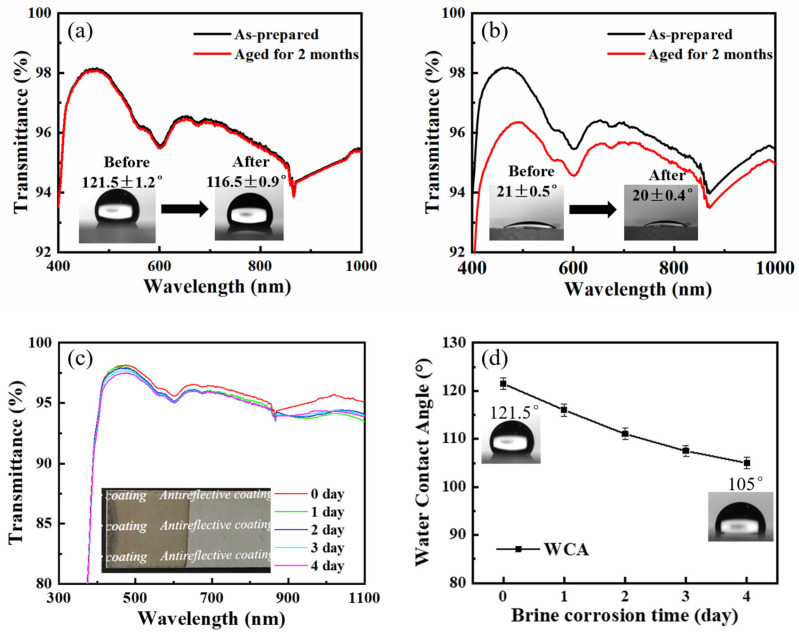
Transmission spectra and water contact angles of the coated PC with (**a**) and without (**b**) HMDS modification after 2 months moist environment test; Transmission spectra (**c**) and WCAs (**d**) of SNs@ACSS-HD coating after immersion in a 0.5 M NaCl solution for varied time.

The environmental resistance of AR coatings is crucial for the practical application of nanocoatings [33,45]. The variation in the transmittance of the coated PC in a humid environment can be used to evaluate the environmental stability of the coatings. Figure 10 shows the change in the transmittance between the SNs@ACSS-HD-coated PC (Figure 10a) and SNs@ACSS-coated PC (Figure 10b) after 2 months at 95% relative humidity at room temperature. After 2 months, the maximum transmittance of the coating without HMDS treatment decreased significantly from 98.18% to 96.36%, and the average transmittance decreased from 96.06% to 95.06% in the wavelength range of 400 to 1000 nm. These results suggest that nanoporous silica coatings are not environmentally stable. In contrast, the transmittance of the SNs@ACSS-HD-coated PC was almost unchanged, and the maximum transmittance only decreased from 98.15% to 98.10%. The above results indicate that the environmental resistance of the AR coating modified by HMDS was significantly improved. The environmental stability of silica AR coatings is determined by the microstructure and surface free energy of the functional groups on the coating surface [46,47]. The methyl groups introduced by HMDS vapor treatment effectively reduced the water adsorption in the humid environment, which improved the environmental resistance of the modified AR coating. Therefore, AR coatings treated with water and HMDS vapor exhibit better environmental stability compared to unmodified coatings.

In this work, the brine corrosion test was also used to evaluate the stability of the coating in a salt solution [29]. Figure 10c,d shows the variations in the transmission spectra and WCAs of the coating before and after corrosion in a 0.5 M NaCl solution. The digital image in Figure 10c demonstrates that the coated PC retained its antireflective property after the brine corrosion test. After 4 days of brine corrosion testing, the average transmittance of the coated PC decreased by only 0.62% in the wavelength range of 400 to 1000 nm. In addition, the coating remained hydrophobic, with a water contact angle of 105°. The above results suggest that the modified AR coating has good resistance to moisture and brine corrosion.

## 4. Conclusions

In summary, we developed a facile method to prepare an AR coating on PC substrates with high transmittance and enhanced mechanical robustness by mixed SNs and ACSS. Excellent antireflective performance with an average transmittance of 96.06% was achieved in the wavelength range of 400 to 1000 nm. SNs provided high transmittance for the coating, and ACSS was utilized, not only as a binder to enhance the adhesion between the PC substrate and the coating but also to significantly improve the mechanical stability of the AR coatings. Meanwhile, water and HMDS vapor treatment significantly increased the hydrophobicity and environmental resistance of the SNs@ACSS coating, and the WCA reached 121.5°. After a series of rigorous tests, the as-prepared AR coating maintained its enhanced transmittance and hydrophobicity. This facile method of preparing high transmittance, mechanically stable and environmentally resistant coatings can be applied to a variety of substrates for different applications, such as optical lenses, shatterproof glass and other optical devices. 

## Data Availability

Not applicable.
